# Supplementary material for: Cyclical dermal micro-niche switching governs the morphological infradian rhythm of mouse zigzag hair
Source: Nat Commun. 2023 Aug 4;14:4478. doi: 10.1038/s41467-023-39605-z (PMC10403492; doi:10.1038/s41467-023-39605-z)
Supplement: Supplementary file 1 — Supplementary information [file 41467_2023_39605_MOESM1_ESM.pdf]

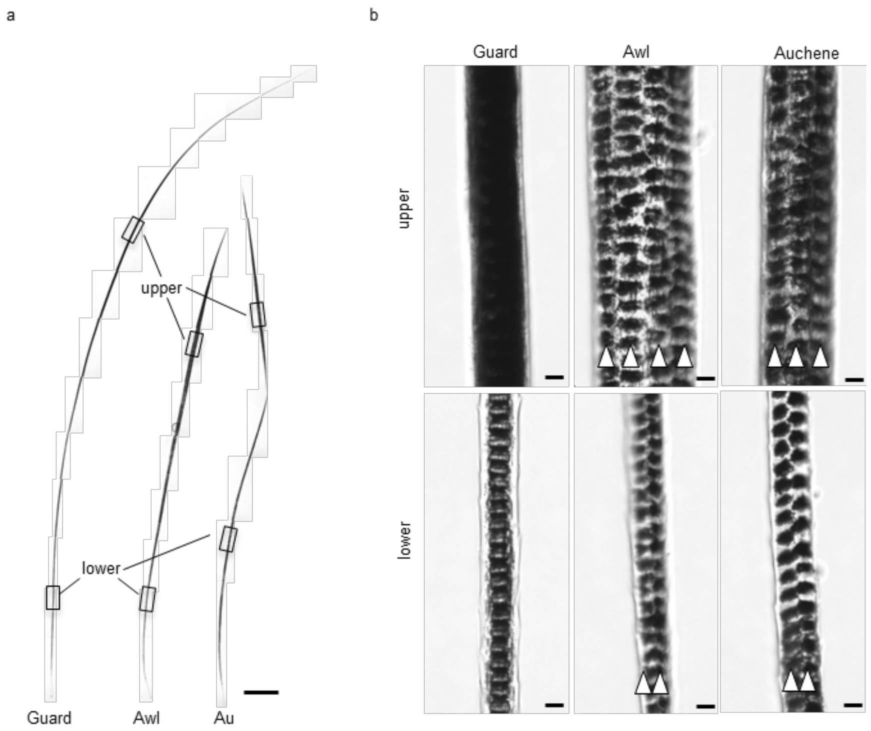

Figure S1. Characterization of guard and awl hair of adult mouse.

a. Shape of guard, awl, and auchene hair shaft in wild-type mice. **b.** Medullar structure of individual hair types at the indicated position in (a). Note that guard and awl hair have no bend, and auchene hair has only one bend. Moreover, awl and auchene hair has several raw medulla cells. Arrowheads indicate the raw medulla cells. The scale bar indicates 1 cm in (a) and 10 μm in (b).

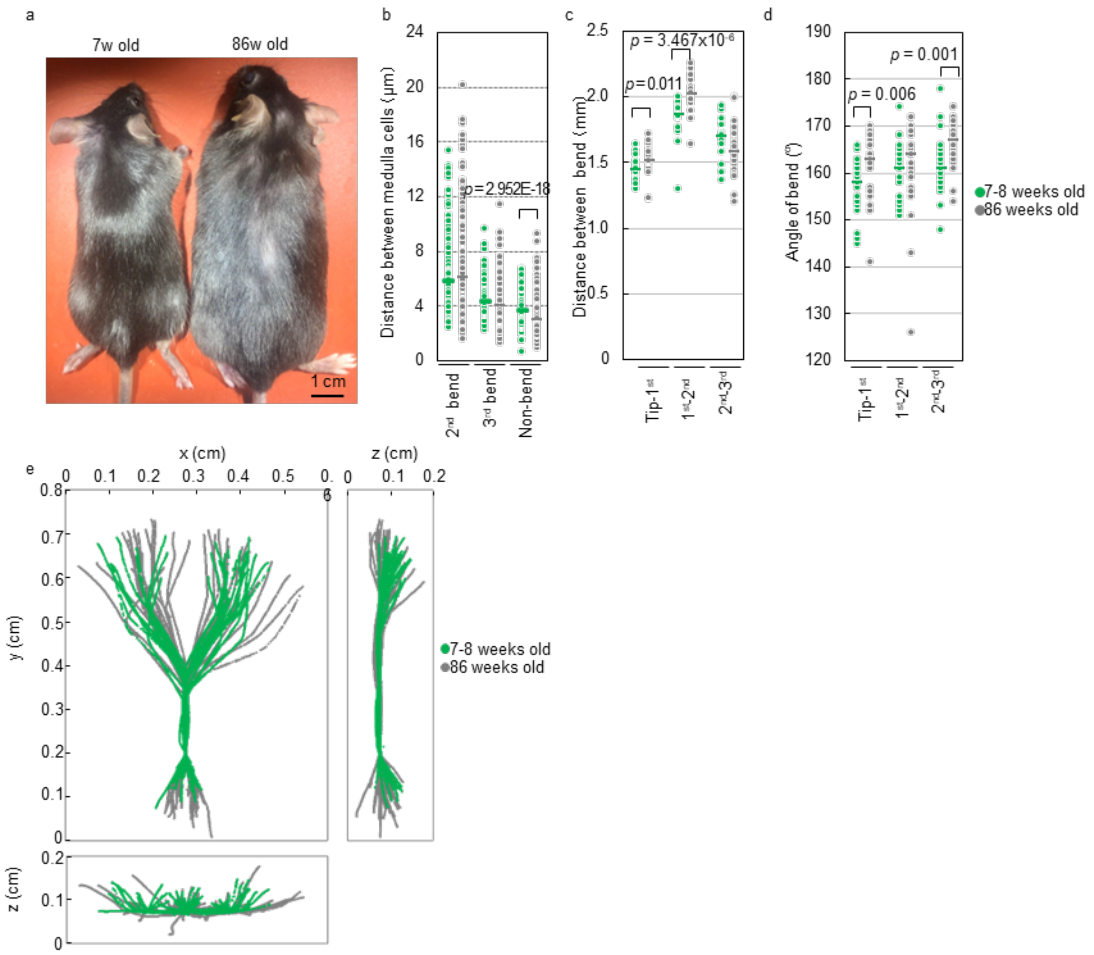

Figure S2. Changes in bend parameters and variations in hair shaft shape occur in aged mice

a. Gross appearance of 7-week-old (left) and 86-week-old (right) wild-type mice. **b.** Quantification of the distance between each medulla cell of zigzag hairs (n = 25 and 32 hair shafts from 3 independent mice at 7 w and 86 w old, respectively). **c.** Quantification of the distance of each bend (n = 29 and 30 hair shafts from 3 independent mice at 7 w and 86 w old, respectively). **d.** Quantification of the angle of each bend (n = 29 and 30 hair shafts from 3 independent mice at 7 w and 86 w old, respectively). **e.** Two-dimensional plot of hair shafts generated from the 3D data shown in (e) (n = 20 hair shafts from 3 independent mice of each age). Quantification data show each data point (circle) and the average value (horizontal bar). ** $p < 0.02$, *** $p < 0.01$ (two-tailed Sudent7s *t*-test). The scale bar indicates 1 cm. Source data are provided as a Source Data file.

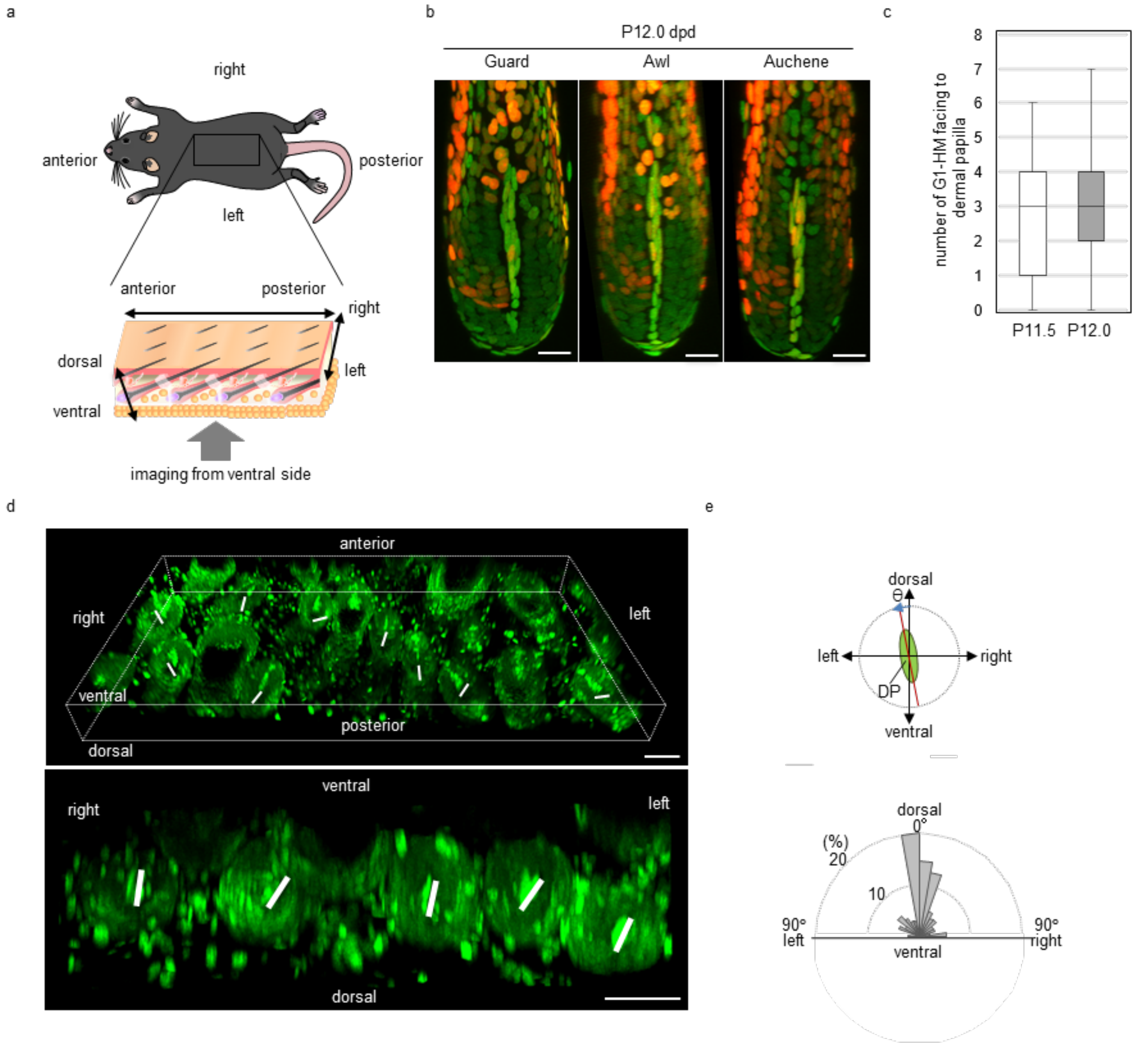

Figure S3. Three-dimensional analysis of the bulb region of multiple hair types. **a.** Experimental schematic. **b.** Analysis of the cell kinetics of the indicated hair type using cell cycle reporter mice at P12.0 dpd. Note the asymmetric distribution of the hair matrix cells in G0/G1 phase. **c.** Quantification of the number of G1-HM cells facing micro-niche C2 shown by box plot with median (horizontal bar) and outlier data points (circle dots) (n = 41 and 45 at P11.5 dpd and P12.0 dpd from at least 3 independent mice, respectively). **d.** Three-dimensional analysis of the angle of the longitudinal axis of the flat plane of dermal papillae. White bars indicate the longitudinal axis of the dermal papilla. **e.** Quantification of the angle of the longitudinal axes (n = 95 from 3 independent mice). Data are shown as box and whisker plots. The bounds of the box plot indicate the 25th and 75th percentiles, the bar indicates medians, the whiskers indicate minima and maxima. Scale bars in (b) and (d) indicate 20 and 50 μm, respectively. Source data are provided as a Source Data file.

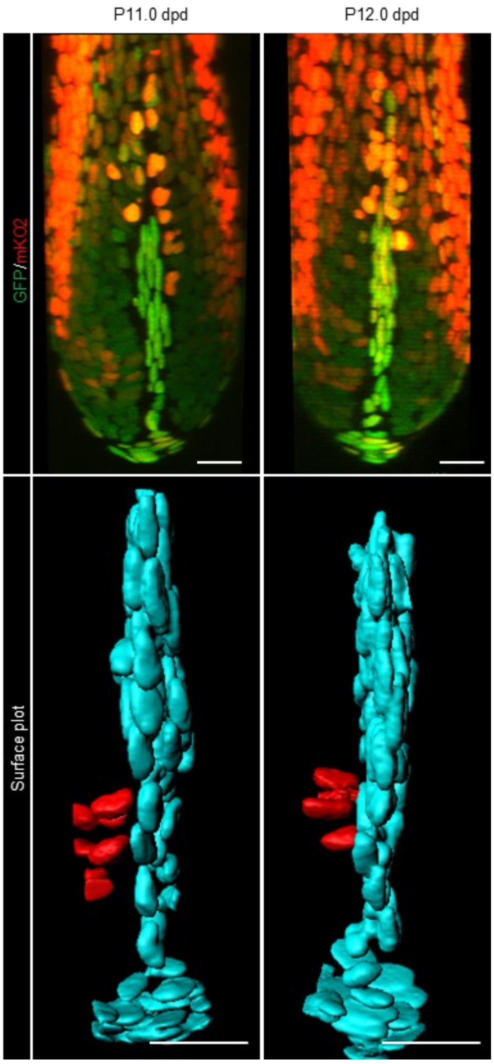

Figure S4. Confocal microscopy and 3D surface plot analysis of awl hair follicles. Analyses were performed at the indicated time points. Three-dimensional surface plots of DP cells were generated from confocal images. Scale bars indicate 20 μm.

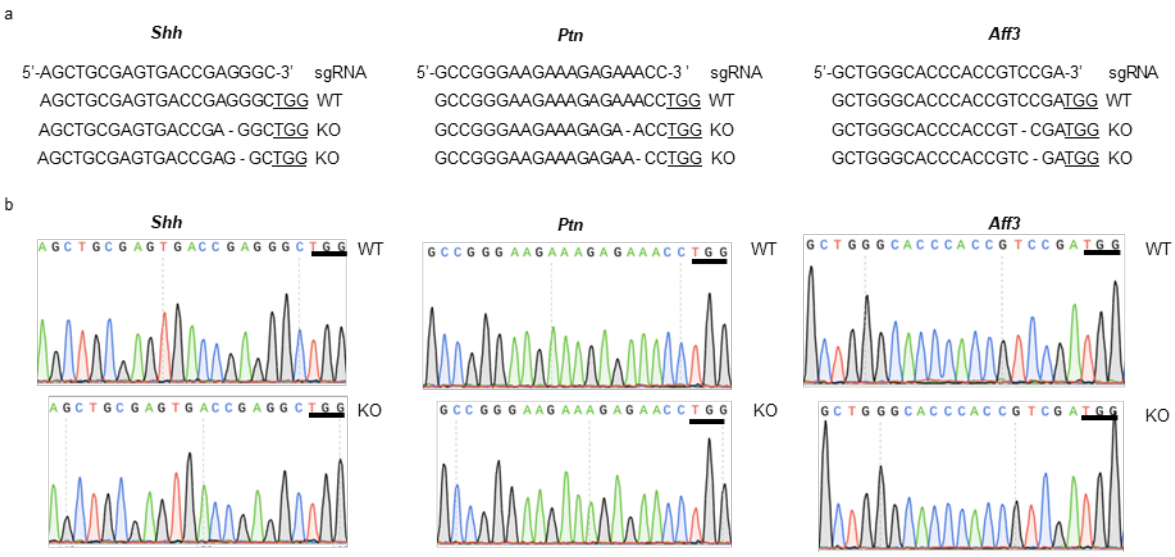

Figure S5. Confirmation of the CRISPR-modified KO allele in regenerated hair follicles.

a. Sequences of sgRNA, wild-type allele (WT), and possible knockout allele (KO) for the indicated genes. **b.** Sequence of regenerated hair follicles. Note that both wild type (WT, upper panel) and knockout allele (KO, lower panel) were detectable.
