# Supplementary material for: Cyclical dermal micro-niche switching governs the morphological infradian rhythm of mouse zigzag hair
Source: Nat Commun. 2023 Aug 4;14:4478. doi: 10.1038/s41467-023-39605-z (PMC10403492; doi:10.1038/s41467-023-39605-z)
Supplement: Supplementary file 3 — Description of Additional Supplementary Files [file 41467_2023_39605_MOESM3_ESM.pdf]

Description of Additional Supplementary Files

File Name: Supplementary Data 1
Description: The information of key materials used in current study

File Name: Supplementary Movie 1
Description: Cross-section images of the hair shaft around the bend

File Name: Supplementary Movie 2
Description: Three-dimensional structure of mouse zigzag hair

File Name: Supplementary Movie 3
Description: Live imaging of the bulb at 12.0 days after hair depilation

File Name: Supplementary Movie 4
Description: Live imaging of the bulb at 13.0 days after hair depilation
