# Supplementary material for: Cyclical dermal micro-niche switching governs the morphological infradian rhythm of mouse zigzag hair
Source: Nat Commun. 2023 Aug 4;14:4478. doi: 10.1038/s41467-023-39605-z (PMC10403492; doi:10.1038/s41467-023-39605-z)
Supplement: Supplementary file 9 — Reporting Summary [file 41467_2023_39605_MOESM9_ESM.pdf]

nature portfolio

Corresponding author(s): Takashi Tsuji

Last updated by author(s): Jun 16, 2023

Reporting Summary

Nature Portfolio wishes to improve the reproducibility of the work that we publish. This form provides structure for consistency and transparency in reporting. For further information on Nature Portfolio policies, see our Editorial Policies and the Editorial Policy Checklist.

Statistics

For all statistical analyses, confirm that the following items are present in the figure legend, table legend, main text, or Methods section.

n/a	Confirmed	
☐	☒	The exact sample size (*n*) for each experimental group/condition, given as a discrete number and unit of measurement
☐	☒	A statement on whether measurements were taken from distinct samples or whether the same sample was measured repeatedly
☐	☒	The statistical test(s) used AND whether they are one- or two-sided<br>*Only common tests should be described solely by name; describe more complex techniques in the Methods section.*
☒	☐	A description of all covariates tested
☒	☐	A description of any assumptions or corrections, such as tests of normality and adjustment for multiple comparisons
☐	☒	A full description of the statistical parameters including central tendency (e.g. means) or other basic estimates (e.g. regression coefficient) AND variation (e.g. standard deviation) or associated estimates of uncertainty (e.g. confidence intervals)
☐	☒	For null hypothesis testing, the test statistic (e.g. *F*, *t*, *r*) with confidence intervals, effect sizes, degrees of freedom and *P* value noted<br>*Give P values as exact values whenever suitable.*
☒	☐	For Bayesian analysis, information on the choice of priors and Markov chain Monte Carlo settings
☒	☐	For hierarchical and complex designs, identification of the appropriate level for tests and full reporting of outcomes
☒	☐	Estimates of effect sizes (e.g. Cohen's *d*, Pearson's *r*), indicating how they were calculated

Our web collection on statistics for biologists contains articles on many of the points above.

Software and code

Policy information about availability of computer code

Data collection	Zen 2.3 SP1 FP1 ver. 14.0.0.0 (Carl Zeiss)
Data analysis	Zen 2012 Blue edition (Carl Zeiss), Imaris x64 ver. 7.6.5, Microsoft Excel Version 2304, RStudio Version 1.4.1106, EdgeR version 3.42.4, ggplot2 version 3.1.3

For manuscripts utilizing custom algorithms or software that are central to the research but not yet described in published literature, software must be made available to editors and reviewers. We strongly encourage code deposition in a community repository (e.g. GitHub). See the Nature Portfolio guidelines for submitting code & software for further information.

Data

Policy information about availability of data

All manuscripts must include a data availability statement. This statement should provide the following information, where applicable:

- Accession codes, unique identifiers, or web links for publicly available datasets
- A description of any restrictions on data availability
- For clinical datasets or third party data, please ensure that the statement adheres to our policy

RNA-seq data set have been deposited in the Gene Expression Omnibus (GEO) database under accession codes GSE211948 (https://www.ncbi.nlm.nih.gov/geo/query/acc.cgi?acc= GSE211948). The datasets generated and analyzed in the current study are available from Source Data file tishi this paper.

Human research participants

Policy information about studies involving human research participants and Sex and Gender in Research.

Reporting on sex and gender	N/A
Population characteristics	N/A
Recruitment	N/A
Ethics oversight	N/A

Note that full information on the approval of the study protocol must also be provided in the manuscript.

Field-specific reporting

Please select the one below that is the best fit for your research. If you are not sure, read the appropriate sections before making your selection.

☒ Life sciences ☐ Behavioural & social sciences ☐ Ecological, evolutionary & environmental sciences

For a reference copy of the document with all sections, see nature.com/documents/nr-reporting-summary-flat.pdf

Life sciences study design

All studies must disclose on these points even when the disclosure is negative.

Sample size	We did not pre-calculate the sample size but chose a statistically reliable sample size. We examine at least 10 samples from each experiment and repeat same experiment at least 3 times. Total sample number is greater than 30, which can detect the phenomenon occurring about 3.3% probability. We think this sample size is enough to calculate significant difference in the phenomenon that we focused in current study.
Data exclusions	We did not exclude any data from the analysis.
Replication	We repeated same experiments at least 3 times and confirm reproducibility.
Randomization	We allocate samples equally and randomly into individual experimental group.
Blinding	We were blinded to sample allocation and during analysis.

Reporting for specific materials, systems and methods

We require information from authors about some types of materials, experimental systems and methods used in many studies. Here, indicate whether each material, system or method listed is relevant to your study. If you are not sure if a list item applies to your research, read the appropriate section before selecting a response.

Materials & experimental systems

n/a	Involved in the study	
☐	☒	Antibodies
☐	☒	Eukaryotic cell lines
☒	☐	Palaeontology and archaeology
☐	☒	Animals and other organisms
☒	☐	Clinical data
☒	☐	Dual use research of concern

Methods

n/a	Involved in the study	
☒	☐	ChIP-seq
☒	☐	Flow cytometry
☒	☐	MRI-based neuroimaging

Antibodies

Antibodies used	Rab Anti-monomeric Kusabira-Orange 2 pAb, MBL, Cat#PM051M, 1:100<br>Anti-hair cortex Cytokeratin/K40 antibody [AE13], abcam, Cat#ab16113, Clone#AE13, 1:50<br>IHC-plus™ Polyclonal Goat anti-Human GATA3 Antibody (aa2-14, IHC, WB) LS-B4879, LifeSpan Biosciences, Cat#LS-B4879, Clone#7B5, 1:100<br>Anti-GATA3 antibody [EPR16651] - ChIP Grade, abcam, Cat#ab199428, Clone#EPR16651, 1:100<br>Biotinylated Horse anti-Goat IgG, VECTOR, Cat#BP-9500, 1:100

	M.O.M. Biotinylated Anti-Mouse IgG Reagent, VECTOR, Cat#BMK-2202 1:100<br>Donkey anti-Rabbit IgG (H+L) Highly Cross-Adsorbed Secondary Antibody, Alexa Fluor 594, ThermoFisher Scientific, Cat#A-21207, 1:200<br>Donkey anti-Mouse Alexa Fluor 647, ThermoFisher Scientific, Cat#A-31571, 1:200<br>Goat anti-Rabbit IgG (H+L) Highly Cross-Adsorbed Secondary Antibody, Alexa Fluor™ 633, ThermoFisher Scientific, Cat#A-21071, 1:200<br>Streptavidin, Alexa Fluor 488 conjugate, ThermoFisher Scientific, Cat#S32354, 1:200<br>Streptavidin, Alexa Fluor™ 405 conjugate, ThermoFisher Scientific, Cat#S32351, 1:200<br>Hoechst 33342, Trihydrochloride, Trihydrate - 10 mg/mL Solution in Water, ThermoFisher Scientific, Cat#H3570<br>Alexa Fluor™ Plus 647 Phalloidin, ThermoFisher Scientific, Cat#A30107, 1:200
Validation	Rab Anti-monomeric Kusabira-Orange 2 pAb (MBL, Cat#PM051M) was validated by MBL on Fucci-G1 Orange transgenic mouse embryonic brain.<br>Anti-hair cortex Cytokeratin/K40 antibody [AE13] (abcam, Cat#ab16113), Anti-GATA3 antibody (abcam, Cat#ab199428) was validated by abcam on mouse skin tissue and human breast carcinoma tissue, respectively.<br>HC-plus™ Polyclonal Goat anti-Human GATA3 Antibody (LifeSpan Biosciences, Cat#LS-B4879) was validated by LifeSpan Biosciences on human spleen tissue

Eukaryotic cell lines

Policy information about cell lines and Sex and Gender in Research

Cell line source(s)	Lenti-X 293T cell line were obtained from TAKARA BIO INC.
Authentication	Cell line used in this study is not authenticated
Mycoplasma contamination	The cell line were not tested for mycoplasma contamination
Commonly misidentified lines (See ICLAC register)	No commonly misidentified cell lines were used.

Animals and other research organisms

Policy information about studies involving animals; ARRIVE guidelines recommended for reporting animal research, and Sex and Gender in Research

Laboratory animals	We used 7-8 weeks old female C57BL/6NCrSlc, BALB/cCrSlc, BALB/cSlc-nu/nu, and H2B-EGFP/Fucci596 reporter mice. We also use embryonic day 18.5 BALB/cCrSlc and H2B-EGFP/Fucci596 reporter mice regardless of the gender. All the experimental procedures using animals were approved by the Institutional Animal Care and Use Committee of RIKEN Kobe Branch and performed in accordance with the relevant guidelines and regulations.
Wild animals	This study did not involve wild animal
Reporting on sex	Sex-based analysis were not performed, because sex does not affect the morphology of the hair shaft, which is the main focus of our study.
Field-collected samples	This study did not involve the samples collected from the field
Ethics oversight	All the experimental procedures using animals were approved by the Institutional Animal Care and Use Committee of RIKEN Kobe Branch

Note that full information on the approval of the study protocol must also be provided in the manuscript.
